# PGC1α Promotes Cisplatin Resistance in Ovarian Cancer by Regulating the HSP70/HK2/VDAC1 Signaling Pathway

**DOI:** 10.3390/ijms22052537

**Published:** 2021-03-03

**Authors:** Yanqing Li, Jinsong Kang, Jiaying Fu, Haoge Luo, Yanan Liu, Yang Li, Liankun Sun

**Affiliations:** Key Laboratory of Pathobiology, Ministry of Education, Department of Pathophysiology, College of Basic Medical Sciences, Jilin University, Changchun 130021, China; yanqingl18@mails.jlu.edu.cn (Y.L.); kangjs@jlu.edu.cn (J.K.); fujy19@mails.jlu.edu.cn (J.F.); luohg19@mails.jlu.edu.cn (H.L.); ynliu@jlu.edu.cn (Y.L.)

**Keywords:** PGC1α, mitochondria, HK2, HSP70, cisplatin, resistance

## Abstract

Mitochondrial apoptosis is one of the main mechanisms for cancer cells to overcome chemoresistance. Hexokinase 2 (HK2) can resist cancer cell apoptosis by expressing on mitochondria and binding to voltage-dependent anion channel 1 (VDAC1). We previously reported that peroxisome proliferator-activated receptor coactivator 1 α (PGC1α) is highly expressed in ovarian cancer cisplatin-resistant cells. However, the underlying mechanism remains unclear. Therefore, we evaluated the interaction between PGC1α and HK2 in ovarian cancer cisplatin-resistant cells. We found that the knockdown of PGC1α promotes the apoptosis of ovarian cancer cisplatin-resistant cells and increases their sensitivity to cisplatin. In addition, we found that the knockdown of PGC1α affects the mitochondrial membrane potential and the binding of HK2 and VDAC1. As the heat shock protein 70 (HSP70) family can help protein transport, we detected it and found that PGC1α can promote HSP70 gene transcription. Furthermore, HSP70 can promote an increase of HK2 expression on mitochondria and an increase of binding to VDAC1. Based on these results, PGC1α may reduce apoptosis through the HSP70/HK2/VDAC1 signaling pathway, thus promoting cisplatin resistance of ovarian cancer. These findings provide strong theoretical support for PGC1α as a potential therapeutic target of cisplatin resistance in ovarian cancer.

## 1. Introduction

Cisplatin resistance is still one of the main causes of death in ovarian cancer patients [1]. Cisplatin resistance can be produced through a variety of mechanisms, such as the cisplatin transport system and the mitochondrial signal pathway [2]. Among them, mitochondria can resist chemotherapy resistance by regulating cell metabolism and apoptosis signals [3,4]. In addition, to cope with the stress caused by chemotherapeutic drugs and to survive, tumor cells use mitochondrial adaptive mechanisms such as mitochondrial biosynthesis and mitochondrial metabolism [5,6]. What is more, studies have demonstrated that mitochondrial biosynthesis-related molecules, such as peroxisome proliferator-activated receptor coactivator 1 α (PGC1α), can regulate apoptosis signaling by interacting with mitochondrial proteins [7,8]. Therefore, targeting mitochondria is a crucial step in preventing the chemotherapy resistance of tumor cells. However, the specific mechanism of mitochondrial resistance to chemotherapy in cisplatin-resistant ovarian cancer cells remains unclear.

As a major regulator of mitochondrial biosynthesis, PGC1α functions in several mitochondrial signaling pathways. For example, the early results of our laboratory found that PGC1α is highly expressed in ovarian cancer cisplatin-resistant SKOV3/CDDP cells compared to ovarian cancer SKOV3 cells [9]. Moreover, downregulation of PGC1α reduces oxidative phosphorylation, promotes apoptosis of non-small cell lung cancer, and induces resistance to cisplatin [10]. In addition, PGC1α inhibits the apoptosis of pancreatic cancer cells by decreasing the expression of anti-apoptotic protein B-cell lymphoma-2 (BCL2) and increasing the expression of pro-apoptotic protein BCL2-associated X (Bax) [11]. Furthermore, PGC1α is involved in the regulation of glucose metabolism. For instance, PGC1α can promote glioblastoma apoptosis by reducing glucose consumption and lactic acid production [12]. However, whether PGC1α can regulate mitochondrial permeability transition pore (MPTP) to induce apoptosis is still unclear.

Hexokinase 2 (HK2) is a defense line to protect mitochondria against apoptosis. The binding of HK2 to voltage-dependent anion channel protein 1 (VDAC1) can maintain the integrity of MPTP, thus inhibiting the apoptosis of hepatocellular carcinoma cells [13]. The stability of MPTP is the main mechanism for cancer cells to resist apoptosis and to promote chemotherapy resistance [14]. Therefore, inhibition of the binding of HK2 and VDAC1 could be a crucial strategy to induce apoptosis of drug-resistant cells. Furthermore, some mitochondrial proteins may regulate the binding of HK2 and VDAC1. For example, mitochondrial glycogen synthase kinase-3β reduces the level of mitochondrial HK2 and apoptosis of melanoma cells by promoting the phosphorylation of HK2 [15]. As the main regulatory molecule of mitochondria, PGC1α is linked to this mitochondrial protein [16]. Therefore, PGC1α may affect the binding of HK2 and VDAC1 by regulating mitochondrial proteins.

Membranes of the heat shock protein (HSP) family or chaperone proteins function to transport proteins to mitochondria [17]. Among them, HSP70, an important member of the HSP family, assists in the transport of J protein and other proteins to the mitochondria [18]. It has been reported that PGC1α reduces the heat stress injury of the liver, kidneys, and fibroblasts of mice by promoting the expression of HSP70 [19,20]. In addition, HSP inhibitors can promote the apoptosis of thyroid cancer cells by reducing glycolysis [21]. Therefore, in this study, we explored the mitochondrial apoptosis mediated by PGC1α through the HSP70/HK2/VDAC1 signaling pathway, which may provide a new strategy for inducing apoptosis of cisplatin-resistant cancer cells.

## 2. Results

### 2.1. Knockdown of PGC1α Promotes the Apoptosis of Cisplatin-Resistant Ovarian Cancer Cells

**Figure 1:**
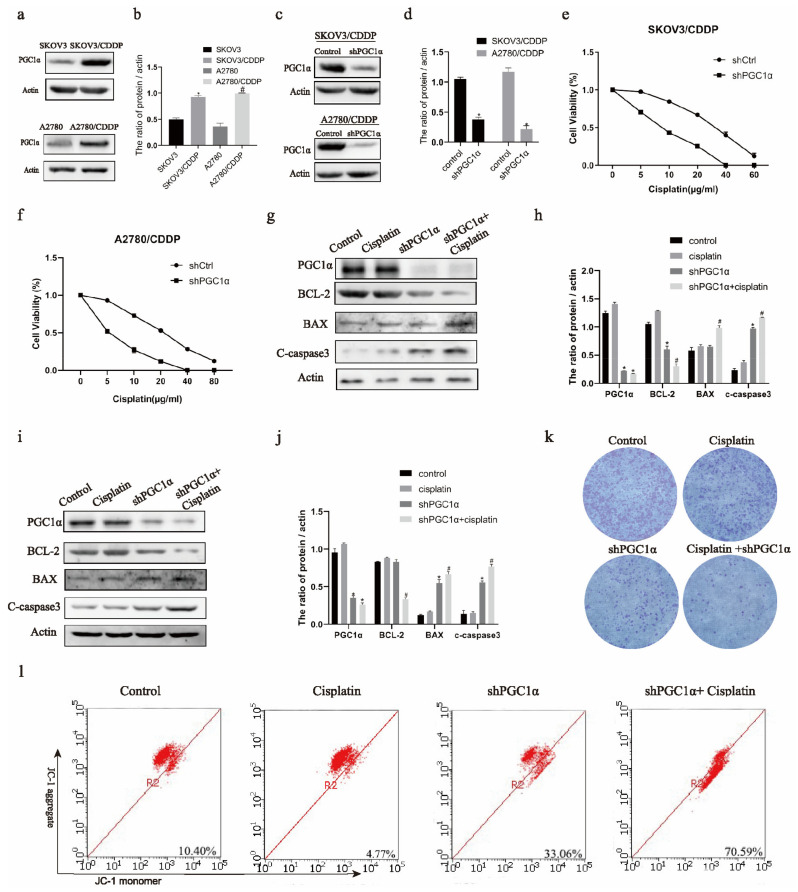
The knockdown of peroxisome proliferator-activated receptor coactivator 1 α (PGC1α) reversed cisplatin resistance in ovarian cancer. Western blot detected PGC1α expression in SKOV3, A2780, SKOV3/CDDP, and A2780/CDDP cells (**a**). The PGC1α protein/actin ratio is expressed as the mean ± SD; *n* = 3, * *p* < 0.05 vs. SKOV3, # *p* < 0.05 vs. A2780 (**b**). The level of PGC1α protein decreased after Western blot detection of shPGC1α (**c**). The protein/actin ratio is expressed as the mean ± SD; *n* = 3, * *p* < 0.05 vs. control (**d**). An MTT assay was used to detect the sensitivity of SKOV3/CDDP (**e**) and A2780/CDDP (**f**) cells to cisplatin with the knockdown of PGC1α and expressed as the mean ± SD; *n* = 3. SKOV3/CDDP cells had high cell viability, and the half maximal inhibitoryconcentration (IC_50)_ was approximately 30 μg/mL, but the IC_50_ after knocking down PGC1α was approximately 5 μg/mL. The A2780/CDDP cells had high cell viability, and the IC_50_ was approximately 22 μg/mL, but the IC_50_ after knocking down PGC1α was approximately 4 μg/mL. Western blot was used to detect B-cell lymphoma-2 (BCL2), BCL2-Associated X (BAX), and cleaved caspase 3 protein expression in SKOV3/CDDP and A2780/CDDP cells (**g**,**i**). The protein/actin ratio is expressed as the mean ± SD; *n* = 3, * *p* < 0.05 vs. control and # *p* < 0.05 vs. cisplatin (**h**,**j**). Clone formation was used to detect the proliferation ability of SKOV3/CDDP cells and is expressed as the mean ± SD; *n* = 3 (**k**). JC-1 was used to detect the mitochondrial membrane potential of SKOV3/CDDP and A2780/CDDP cells and expressed as the mean ± SD; *n* = 3 (**l**).

Western blotting showed increased expression of PGC1α in ovarian cancer cisplatin-resistant SKOV3/CDDP and A2780/CDDP cells than in ovarian cancer SKOV3 and A2780 cells (Figure 1a,b). Therefore, we knocked down PGC1α in SKOV3/CDDP and A2780/CDDP cells (Figure 1c,d). Additionally, the MTT assay showed that SKOV3/CDDP and A2780/CDDP cells were more sensitive to cisplatin after PGC1α knockdown (Figure 1e,f). In addition, PGC1α-knockdown cells underwent apoptosis (Figure 1g–j). Western blotting showed that the level of anti-apoptotic protein BCL-2 reduced after the knockdown of PGC1α and the addition of cisplatin, whereas that of pro-apoptotic protein BAX and c-caspase 3 increased in SKOV3/CDDP and A2780/CDDP cells (Figure 1g–j). Clonal formation showed that the proliferation of SKOV3/CDDP cells decreased after knocking down PGC1α and adding cisplatin at the same time (Figure 1k). Finally, the JC1 assay showed that the mitochondrial membrane potential decreased and the sensitivity to cisplatin increased after PGC1α knockdown in SKOV3/CDDP and A2780/CDDP cells (Figure 1l).

### 2.2. HK2 Is a Key Molecule in MPTP of Cisplatin Resistance in Ovarian Cancer and Is Mediated by PGC1α

**Figure 2:**
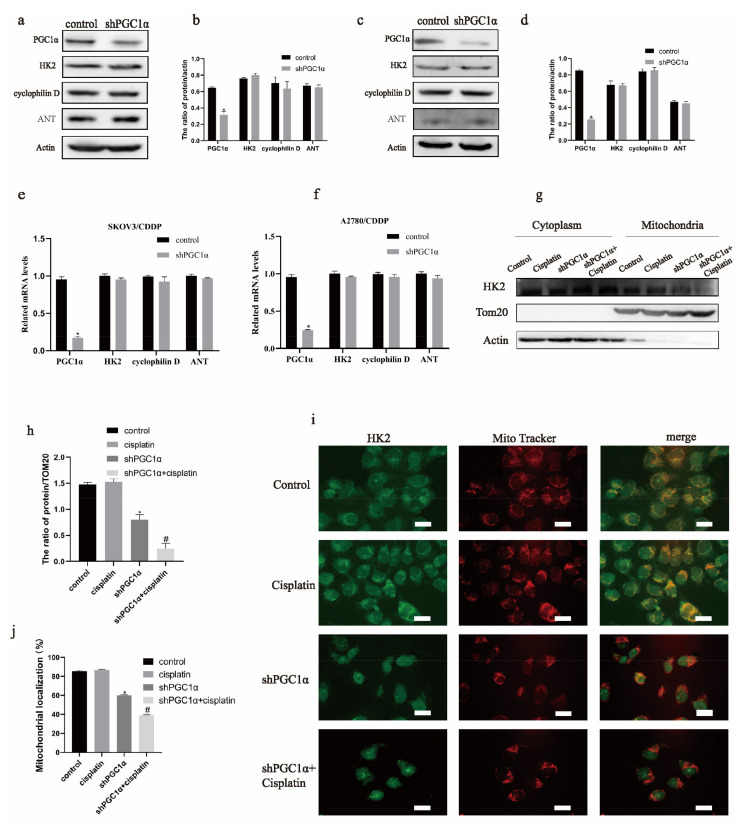
PGC1α changed the mitochondrial expression of hexokinase 2 (HK2) in ovarian cancer cisplatin-resistant cells. After knockdown PGC1α, Western blot was used to detect the protein expressions of HK2, cyclophilin D, and adenine nucleotide translocase (ANT) in SKOV3/CDDP and A2780/CDDP cells (**a**,**c**). The protein/actin ratio is expressed as the mean ± SD; *n* = 3, * *p* < 0.05 vs. control (**b**,**d**). Quantitative real-time PCR (qPCR) detected the mRNA expression of HK2, cyclophilin D, and ANT in SKOV3/CDDP and A2780/CDDP cells (**e**,**f**), expressed as the mean ± SD; *n* = 3, * *p* < 0.05. A mitochondrial extraction kit was used to detect HK2 protein expression in the mitochondria of SKOV3/CDDP cells (**g**). The protein/TOM20 ratio is expressed as the mean ± SD; *n* = 3, * *p* < 0.05 vs. control and # *p* < 0.05 vs. cisplatin (**h**). An immunofluorescence assay was used to detect the localization of HK2 in mitochondria in A2780/CDDP cells (**i**). The percentage of mitochondrial localization was analyzed by ImageJ and expressed as the mean ± SD; *n* = 3, * *p* < 0.05 vs. control and # *p* < 0.05 vs. cisplatin (**j**).

Since the knockdown of PGC1α reduced the mitochondrial membrane potential in ovarian cancer cisplatin-resistant cells, we targeted the mitochondrial permeability transition pore. As shown in Figure 2a–f, no significant differences in the protein and mRNA levels of HK2, adenine nucleotide translocase (ANT), and cyclophilin D were found following PGC1α knockdown. Since the mitochondrial localization of HK2 affects the opening of MPTP, we detected the mitochondrial expression of HK2. Furthermore, mitochondrial extraction showed that the mitochondrial expression of HK2 protein decreased after PGC1α knockdown and the addition of cisplatin (Figure 2g,h). In addition, immunofluorescence revealed that the localization of HK2 to the mitochondria decreased in A2780/CDDP cells (Figure 2i,j). These results indicate that HK2 could be involved in regulating PGC1α-induced cisplatin resistance in ovarian cancer cells.

### 2.3. Knockdown of PGC1α Affects the Binding of HK2 to VDAC1 in Ovarian Cancer Cisplatin-Resistant Cells

**Figure 3:**
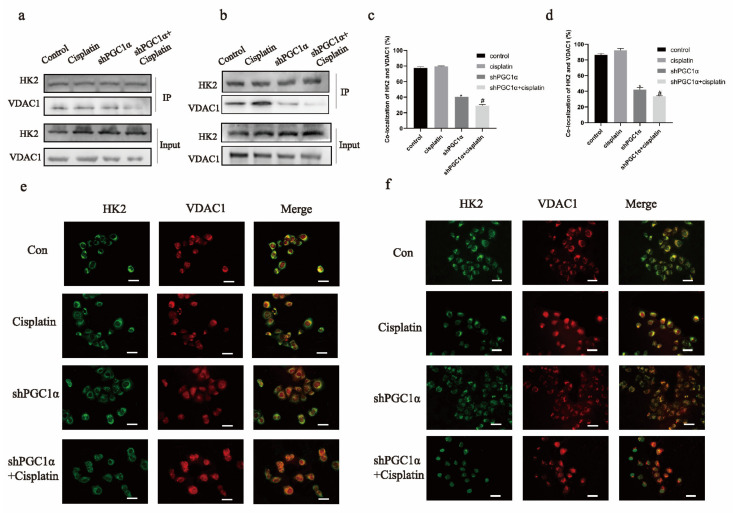
The knockdown of PGC1α reduced the binding of HK2 and VDAC1 in ovarian cancer cisplatin-resistant cells. An immunoprecipitation assay was used to detect the binding of HK2 to VDAC1 in SKOV3/CDDP and A2780/CDDP cells and expressed as the mean ± SD; *n* = 3 (**a**,**b**). An immunofluorescence assay was used to detect the binding of HK2 to VDAC1 in SKOV3/CDDP and A2780/CDDP cells (**e,f**). The percentage of HK2 and VDAC1 localization was analyzed by ImageJ and expressed as the mean ± SD; *n* = 3, * p < 0.05 vs. control and *# p* < 0.05 vs. cisplatin (**c,d**).

HK2 binds to VDAC1 in the mitochondria to prevent apoptosis in cancer cells. Thus, we tested the binding of HK2 to VDAC1. Immunoprecipitation showed that the binding of HK2 to VDAC1 decreased after PGC1α was knocked down and cisplatin was added simultaneously in SKOV3/CDDP and A2780/CDDP cells (Figure 3a,b). Moreover, the immunofluorescence results showed that the co-localization of HK2 and VDAC1 was decreased in SKOV3/CDDP and A2780/CDDP cells (Figure 3c–f).

### 2.4. HSP70 Assists PGC1α to Regulate the Mitochondrial Function of HK2 in Ovarian Cancer Cisplatin-Resistant Cells

**Figure 4:**
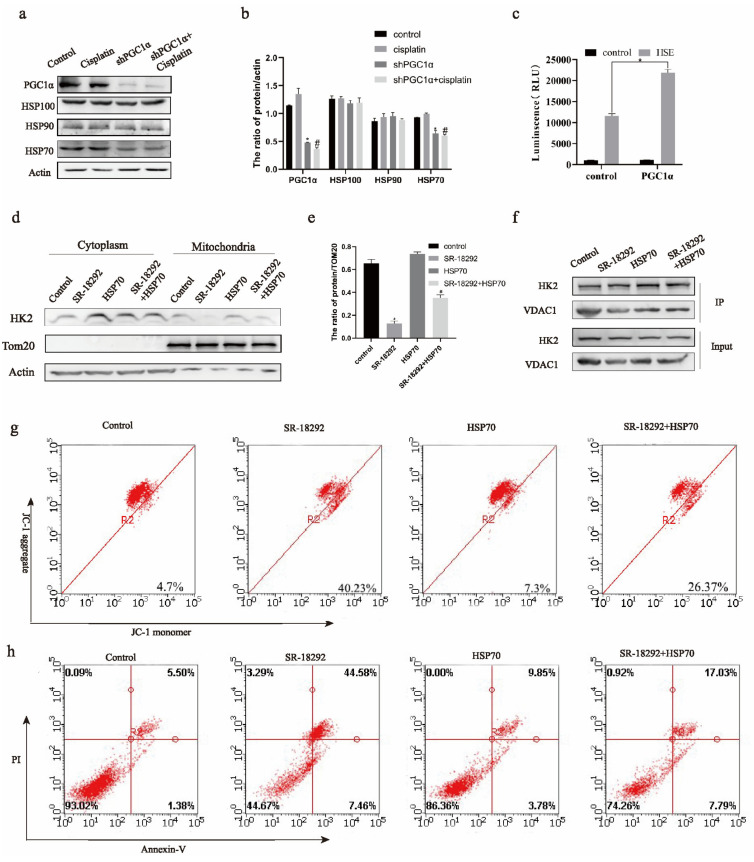
PGC1α regulates the function of HK2 in mitochondria through heat shock protein 70 (HSP70). The protein expressions of HSP100, HSP90, and HSP70 were detected by Western blot in SKOV3/CDDP cells (**a**). The protein/actin ratio is expressed as the mean ± SD; *n* = 3, * *p* < 0.05 vs. control and # *p* < 0.05 vs. cisplatin (**b**). The luciferase reporter gene was used to detect whether PGC1α can promote the transcription of HSP70 promoter heat shock factor 1 (HSF1) and expressed as the mean ± SD; *n* = 3, * *p* < 0.05 vs. control (**c**). After PGC1α inhibitor SR-19282 and overexpressed HSP70 at the same time, the mitochondria expressions of HK2 (**d**) were detected. The protein/ translocase of outer mitochondrial membrane 20 (TOM20) ratio is expressed as the mean ± SD; *n* = 3, * *p* < 0.05 vs. control and # *p* < 0.05 vs. SR-18292 (**e**). After PGC1α inhibitor SR-19282 and overexpressed HSP70 at the same time, the combination HK2–VDAC1 in SKOV3/CDDP cells was detected (**f**). After PGC1α inhibitor SR-19282 and overexpressed HSP70 at the same time, the mitochondrial membrane potential (**g**) and apoptosis (**h**) in SKOV3/CDDP cells were detected and expressed as the mean ± SD; *n* = 3.

Members of the HSP family function to transport proteins to mitochondria. Western blotting showed that the expression of HSP70 protein decreased after the addition of cisplatin and the knockdown of PGC1α in SKOV3/CDDP cells (Figure 4a,b). Therefore, we explored the relationship between HSP70 and HK2. Therefore, we explored the relation between PGC1α and HSP70. The luciferase assay showed that PGC1α could be a transcription cofactor of HSP70 promoter HSF1 (Figure 4c). The expression of mitochondrial HK2 increased (Figure 4d,e) in SKOV3/CDDP cells when PGC1α was inhibited and HSP70 was overexpressed simultaneously. Furthermore, immunoprecipitation showed that HSP70 could bind to HK2 in SKOV3/CDDP cells (Figure 4e). In addition, the binding of HK2 to VDAC1 increased (Figure 4f) in SKOV3/CDDP cells when PGC1α was inhibited and HSP70 was overexpressed simultaneously. Finally, the mitochondrial membrane potential and apoptosis decreased (Figure 4g,h) in SKOV3/CDDP cells when PGC1α was inhibited and HSP70 was overexpressed simultaneously. Based on these results, we conclude that PGC1α can regulate HSP70 transcription. Furthermore, HSP70 induces the expression of HK2 in mitochondria and its binding with VDAC1, consequently promoting cisplatin resistance in ovarian cancer.

### 2.5. PGC1α Manages Mitochondrial Function of HK2 through SBD Domain of HSP70 in Ovarian Cancer Cisplatin Resistant Cells

**Figure 5:**
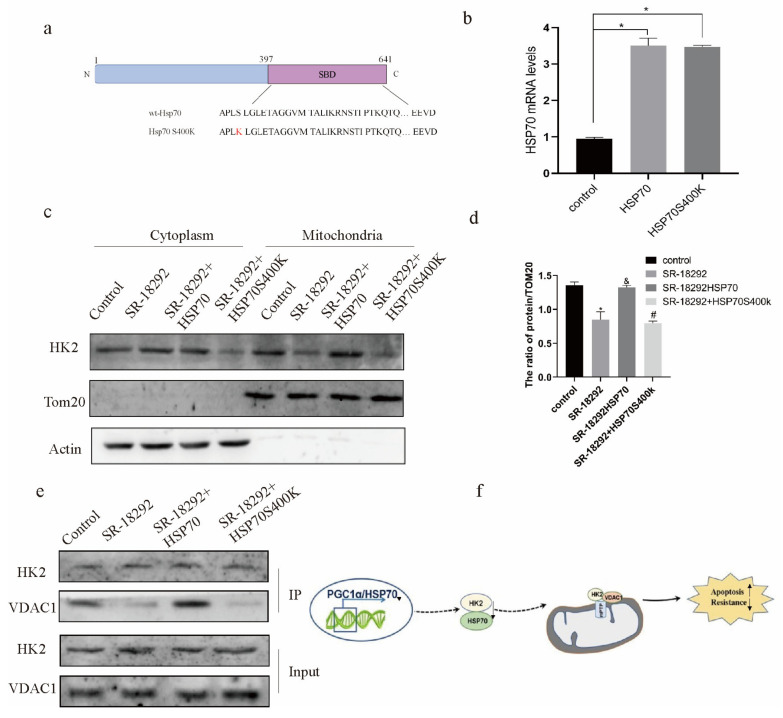
PGC1α manages the mitochondrial expression of HK2 and glycolysis through HSP70. We mutated serine at 400 sites of HSP70 into lysine (**a**). The mRNA level of HSP70 was verified by qPCR and expressed as the mean ± SD; *n* = 3, * *p* < 0.05 vs. control (**b**). The effect of the overexpression of HSP70 and HSP70 S400K on the expression level of HK2 mitochondria was detected by a mitochondrial extraction experiment (**c**). The protein/TOM20 ratio is expressed as the mean ± SD; *n* = 3, * *p* < 0.05 vs. control, & *p* < 0.05 vs. SR-18292 and *# p* < 0.05 vs. SR-18292+HSP70 (**d**). Immunoprecipitation was used to detect the binding of HK2 to VDAC1 after SKOV3/CDDP cells were treated and expressed as the mean ± SD; *n* = 3 (**e**). Therefore, we speculate that PGC1 regulates the binding of HK2 to VDAC1 through HSP70, and then affects the cisplatin resistance of ovarian cancer (**f**).

HSP70 has a substrate binding domain (SBD), which can help protein refolding and protein transport. Therefore, we performed an amino acid mutation at the 400 sites of HSP70 (Figure 5a,b). When PGC1α was inhibited and HSP70 was overexpressed, the mitochondrial expression of HK2 was promoted. However, when the SBD domain of HSP70 was mutated, the expression of HK2 in the mitochondria decreased (Figure 5c,d). In addition, when the SBD domain of HSP70 was mutated, the binding of HK2 to VDAC1 was decreased in SKOV3/CDDP cells (Figure 5e). Therefore, PGC1α may promote cisplatin sensitivity of ovarian cancer cells through the HSP70/HK2/VDAC1 signaling pathway (Figure 5f).

## 3. Discussion

Ovarian cancer ranks fifth among all malignant tumors and is a major gynecological tumor that results in the death of women [22]. At present, the treatment of ovarian cancer involves a comprehensive approach based on radical operation followed by platinum drugs [23,24]. However, several patients become resistant to chemotherapeutic drugs during chemotherapy, significantly reducing their five-year survival rate [25]. Therefore, understanding the mechanism of the drug resistance of tumor cells and potential therapeutic drugs is of utmost importance. We previously reported that PGC1α is highly expressed in cisplatin-resistant ovarian cancer cells, suggesting that PGC1α might be related to cisplatin resistance in ovarian cancer [9]. However, it is still unclear how PGC1α promotes cisplatin resistance in ovarian cancer. In the present study, we reported a new signaling pathway involving PGC1α that promotes cisplatin resistance in epithelial ovarian cancer cells.

PGC1α is linked to the drug resistance of cancer cells. In chemotherapy of colon cancer, the PGC1α signaling pathway upregulates the mitochondrial respiratory complex protein and enhances the oxygen consumption rate, thus promoting drug resistance [26]. In addition, PGC1α elevates the mitochondrial oxidative phosphorylation in non-small cell lung cancer cisplatin-resistant cells to resist reactive oxygen species (ROS) damage caused by cisplatin and promotes the drug resistance of cancer cells [10]. Increased ROS production in cancer cells directly acts on mitochondrial membrane proteins, resulting in increased mitochondrial outer membrane permeability, loss of mitochondrial membrane potential, and apoptosis [27]. We found that compared to ovarian cancer cells, those resistant to cisplatin had a higher expression level of PGC1α. Therefore, after we knocked down PGC1α, the mitochondrial membrane potential of cisplatin-resistant ovarian cancer cells decreased and apoptosis increased. Our findings promoted the correlation between PGC1α and mitochondrial membrane protein, and provided a new idea for the mechanism of PGC1α and apoptosis.

MPTP plays a pivotal role in resisting apoptosis in cancer cells. Previous studies have found that mitochondrial HK2, an important member of MPTP, can exchange ATP/ADP by binding with VDAC and maintaining its open configuration, thereby preserving the integrity of MPTP and preventing apoptosis [28]. Thus, an increase in mitochondrial HK2 may promote the stability of MPTP. It was previously found that compared to ovarian cancer cells, the expression of HK2 in cisplatin-resistant ovarian cancer cells increases [29,30]. However, this phenomenon has not been explained in MPTP. We found that after infection with shPGC1α and the simultaneous addition of cisplatin, the mitochondrial localization of HK2 and the binding of HK2 and VDAC1 in ovarian cancer drug-resistant cells decreased and apoptosis increased. This explains that targeting mitochondria can greatly reduce the cisplatin resistance of ovarian cancer cells. The regulatory relationship between PGC1α and mitochondrial MPTP was discovered for the first time, which added a new pathway to the signal network of cisplatin-resistant ovarian cancer cells. However, the mechanism of how PGC1α affects the binding between HK2 and VDAC1 needs to be further explored.

Members of the HSP family function to transport proteins. It has been found that PGC1α binds to HSF1 and promotes its transcription both in vivo and in vitro. Moreover, our results demonstrated that PGC1α can promote the transcription of HSF1 [19]. After the transfection of cells with shPGC1α, the expression of HSP70, a downstream transcription factor of HSF1, decreased. HSP70 can help transport proteins with an N-terminal helix structure to the mitochondrial outer membrane [18,31]. HK2 is a mitochondrial outer membrane protein with an N-terminal helix structure; however, it has not been reported whether HSP70 is involved in its transport. After PGC1α was knocked down, the protein level of HSP70 decreased. In addition, immunoprecipitation showed that the binding of HK2 and VDAC1 decreased. Therefore, we used inhibitors of PGC1α and overexpressed HSP70 at the same time. The results showed that the binding of HK2 to VDAC1 and the mitochondrial membrane potential increased. Therefore, PGC1α may influence the binding of HK2 and VDAC1 by regulating HSP70. We continued to mutate the HSP70SBD domain, and compared with the previous step, we found that the binding between HK2 and VDAC1 decreased. Our results indicate that PGC1α may regulate the binding between HK2 and VDAC1 through the SBD domain of HSP70. This provides a new mechanism for PGC1α to regulate the mitochondria and for its involvement in cisplatin resistance in ovarian cancer.

In conclusion, PGC1α can regulate the binding between HK2 and VDAC1 through the SBD domain of HSP70. This affects the stability of MPTP and further affects the process of cisplatin resistance in ovarian cancer. Our findings provide a new mechanism for PGC1α to regulate cisplatin resistance in mitochondria and ovarian cancer. It also provides theoretical support for the clinical application of the PGC1α inhibitor SR-18292. In addition, it also provides a new idea for screening cancer biomarkers. In clinical practice, we should not only observe the expression differences of molecules, but also pay attention to the subcellular localization of molecules. As PGC1α is also involved in cell glucose metabolism and oxidative phosphorylation, we will continue to explore the mechanism of PGC1α in glucose transport, glycolysis, and oxidative phosphorylation. In addition, due to the limitation of cell lines, our research will increase HGSOC cell lines and ovarian cancer tissues in the future.

## 4. Materials and Methods

### 4.1. Cell Culture

The A2780, SKOV3, A2780/CDDP, and SKOV3/CDDP cells were acquired from the Chinese Academy of Medical Sciences (Beijing, China). Cells were cultured in RPMI-1640 medium (Gibco, Grand Island, NY, USA) in an incubator (Thermo Fisher, Waltham, MA, USA) at 37 °C and in 5% CO_2_.

### 4.2. Plasmid Transfection

The shRNA sequence and empty sequence of human PGC1α were constructed by GeneChem Co., Ltd. (Shanghai, China). The PGC1α shRNA sequences were 5′-GTTATACCTGTGATGCTTT-3′ and 5′-CAGCGAAGATGAAAGTGA-T-3′ and the non-target shRNA (scramble) sequence was 5′-TTCTCCAAGTGTCAGT-3′. Plasmid-overexpressing human HSP70 was constructed by the Public Protein/Plasmid Library company (Beijing, China). The plasmids were transfected using the TurboFect transfection reagent (Thermo Fisher Scientific, Waltham, MA, USA). According to the manufacturer’s instructions, the cells were spread in a 6-well plate and were transfected with 4 g plasmid and 6 µL transfection reagent the next day. For subsequent experiments, cells were collected 24 h after transfection.

### 4.3. Cell Viability Detection

The cells were inoculated into a 96-well plate (8 × 103 cells/well). During logarithmic growth, the cells were treated with cisplatin and plasmid, and MTT was added after 24 h. After 4–6 h, the absorbance at a wavelength of 570 was detected using a microplate reader (Bio-Rad, California, CA, USA).

### 4.4. Western Blotting

The protein samples were subjected to 12% (*w*/*v*) sodium dodecyl sulfate (SDS)–polyacrylamide gel electrophoresis to separate proteins of different molecular weights. Electrophoresis was performed at 100 V for 100 min. Proteins were transferred to PVDF membranes at 100 V for 2 h. In addition, 5% (*w*/*v*) skim milk powder was used for blocking the membranes at room temperature for 1.5–2 h and the corresponding antibody was added overnight at 4 °C. Finally, after incubation with the secondary antibody for 1.5–2 h at room temperature, protein bands were imaged using a gel imager (Syntyos, Cambridge, MA, USA). Antibodies against PGC1α (66369-1-Ig), HK2 (66974-1-Ig), VDAC1 (66345-1-Ig), BCL-2 (12789-1-AP), BAX (50599-2-Ig), Actin (23660-1-AP), TOM20 (11802-1-AP), and HSP70 (13174-1-AP) were purchased from Proteintech (Chicago, IL, USA). Antibody against cleaved caspase 3 (ab32042) was procured from Abcam (Cambridge, UK).

### 4.5. Immunofluorescence

Cells were seeded on coverslips (5 × 104 cells/well) in 24-well plates overnight and pretreated under different conditions. The cells were fixed with 4% (*w*/*v*) paraformaldehyde for 20 min, stained with the first antibody for 3 h, followed by staining with the second antibody for 30 min. The coverslips were finally sealed with glycerol. Images were captured using the Olympus FV1000 confocal laser microscope (Tokyo, Japan).

### 4.6. Real-Time Fluorescent Quantitative PCR

All primers for real-time fluorescent quantitative PCR (RT-qPCR) were constructed by Sangon Biotech (Beijing, Shanghai). PGC1α: 5′-TCTGAGTCTGTATGGAGTGACAT-3′ (forward primer), 5′-CCAAGTCGTTCACATCTAGTTCA-3′ (reverse primer); HK2: 5′-GAGCCACCACTCACCCTACT-3′ (forward primer), 5′-CCAGGCATTCGGCAATGTG-3′ (reverse primer); ANT: 5′-TTGCCTCCCAACATAAAAGACAG-3′ (forward primer), 5′-GAAGTTACAGAAACTCGGTTCCC-3′ (reverse primer); Cyclophilin D: 5′-GAAGTTACAGAAACTCGGTTCCC-3′ (forward primer), 5′-GTGTGCCCCAAAACATGCG-3′ (reverse primer); Actin: 5′-TTTCTGAGTTGATTTCCCGGTC-3′ (forward primer), 5′-ACCGAACTTGCATTGATTCCAG-3′ (reverse primer). The total RNA of the cells was extracted according to the instructions provided in the TRIzol kit (Invitrogen, Carlsbad, CA, USA). Next, cDNA was synthesized by reverse transcription according to the instructions given in the RT2 First Strand kit (Promega, Madison, MI, USA). Finally, the assay was performed on the CFX96 Touch Real Time PCR Detection System (Bio-Rad Laboratories, Inc.). The relative expression between different experimental groups was calculated by the 2-ΔΔCq method and normalized to the expression of Actin.

### 4.7. Immunoprecipitation

The pretreated samples were incubated with antibody and protein A and G agarose beads (Beyotime Biotechnology, Shanghai, China) for 24 h. The protein sample was extracted from the supernatant after centrifugation. The protein sample was analyzed by Western blotting.

### 4.8. Flow Cytometry

The pretreated cell samples were incubated for 30 min in 1 mL of complete medium containing 10 mg/mL of JC-1 (Beyotime Biotechnology). The cell mitochondrial membrane potentials were analyzed by running the samples on Accuri C6 flow cytometer (BD Biosciences, Franklin Lakes, NJ, USA) and incubated in a solution containing phycoerythrin and Annexin V (Beyotime Biotechnology) for 30 min. Apoptosis was studied by Accuri C6 flow cytometry (BD Biosciences).

### 4.9. Mitochondrial Extraction

The mitochondria were isolated from the pretreated samples using a minute (tm) mitochondrial isolation kit (Invent Biotechnology Inc., Plymouth, MN, USA). The isolated mitochondria were dissolved in 0.5% (*v*/*v*) Triton x-100 and detected using Western blotting. The antibodies against PGC1α (66369-1-Ig), HK2 (66974-1-Ig), and Tom20 (11802-1-AP) were purchased from Proteintech (Chicago, USA).

### 4.10. Luciferase Report Gene Detection

The HSF1 luciferase reporter gene was obtained from Saixin Biotechnology Co., Ltd. (Changchun, China). The firefly luciferase reporter gene detection kit was obtained from Beyotime (RG005, Shanghai, China). The PGC1α overexpression plasmid and HSF1 luciferase reporter gene plasmid were transfected into ovarian cancer resistant SKOV3/CDDP cells. The cells were collected after 24 h and treated as per the instructions given in the firefly luciferase reporter gene detection kit. The chemiluminescence was detected using a microplate reader.

### 4.11. Clone Formation

The cells of each experimental group were digested with pancreatin, and then added into the complete culture medium for suspension. The cells were inoculated into 12-well plates with 250 cells per well and cultured for 9 days. Pictures were taken under a microscope (Tokyo, Japan).

### 4.12. Statistical Analysis

A Mann–Whitney test was used to compare the data between the two groups. The Kruskal–Wallis test was used for multiple comparisons. A *p* < 0.05 was considered significant.

## 5. Conclusions

We found that PGC1α regulates mitochondrial apoptosis and glycolysis through the HSP70/HK2 pathway to promote cisplatin resistance in ovarian cancer cells. Thus, PGC1α could be a potential therapeutic target of cisplatin resistance in ovarian cancer. However, owing to the large number of HSP70 subtypes, it is still unclear which subtype plays a major role. We plan to address this in our future work. Moreover, we will study the mechanism of cisplatin resistance in ovarian cancer tissues.

## Data Availability

All data are available.
